# Community treatment orders and reduced time in hospital: a nationwide study, 2007–2012

**DOI:** 10.1192/pb.bp.115.051045

**Published:** 2016-06

**Authors:** Mark Taylor, Melanie Macpherson, Callum Macleod, Donald Lyons

**Affiliations:** 1 Royal Edinburgh Hospital, UK; 2 University of Edinburgh, UK; 3 University of Queensland, Australia; 4 Mental Welfare Commission for Scotland, Edinburgh, UK

## Abstract

**Aims and method** Community treatment orders (CTOs) were introduced in Scotland in 2005, but are controversial owing to a lack of supportive randomised evidence. The non-randomised studies provide mixed results on their efficacy and utility. We aimed to examine hospital bed day usage across Scotland both before and after CTOs were initiated in a national cohort of patients, spanning 5 years.

**Results** In total, 1558 individuals who were subject to a CTO between 2007 and 2012, of whom 63% were male, were included. After CTO initiation the number of hospital bed days fell, on average, from 66 to 39 per annum per patient. Those with a longer psychiatric history appeared to benefit more from a CTO, in terms of reduced time in hospital.

**Clinical implications** Our data offer cautious support for the use of CTOs in routine practice, in terms of reducing time spent in psychiatric hospital. This finding is balanced by the more rigorous randomised studies which do not find any benefit to CTOs.

Community treatment orders, known as CTOs in England and Wales and CCTOs (community compulsory treatment orders) in Scotland, were specifically legislated under the relevant mental health acts to reduce repeated hospital admissions and promote patient autonomy and independent living. However, the lack of supportive randomised scientific evidence related to efficacy,^1^ and in particular a negative randomised controlled trial (RCT) from Oxford,^2^ has led to considerable debate on their use.^3^ A survey of psychiatrists in England and Wales found that the majority felt CTOs would be therapeutically useful,^4^ although a recent systematic review from the Oxford team noted contradictory results from the non-randomised data.^5^ Nevertheless, the implementation of CTOs has been rapid in the UK, for example they now represent 30% of all long-term treatment orders in Scotland, where they have been in use since 2005.^6^

Here we examine the effect of CTOs on hospital bed use in Scotland, hypothesising that there would not be any relationship between use of a CTO and number of hospital bed days.

## Method

All individuals subject to a CTO in Scotland between 2007 and 2012 were identified from the Mental Welfare Commission for Scotland database. The Commission is a statutory body overseeing the rights and welfare of people with a mental disorder, and routinely collects information on all individuals detained under the Mental Health (Care and Treatment) (Scotland) Act 2003. Information on each person subject to a CTO was anonymised prior to analysis, and included gender, age at which the person was placed on a CTO, diagnosis and involuntary hospital admissions before, during and, if applicable, after a CTO until 7 March 2013. Diagnostic data were limited to the simple categories of mental illness, personality disorder or intellectual disability.

An analysis of time spent in hospital before and after CTO institution was conducted, for both *de novo* CTOs (i.e. no prior Mental Health (Care and Treatment) (Scotland) Act detention) and for those people on CTOs who had been on varied hospital-based orders immediately beforehand. Time before and after CTO use varied for each individual, and depended on date of first detention. To aid comparison the average number of days spent in hospital per year, before and after the CTO was instituted, was calculated.

Quantitative analysis was then performed using IBM SPSS Statistics (v11 for Windows), with a related-samples *t*-test being used to compare the mean number of days in hospital per year before and after a CTO. An independent-samples *t*-test was used to compare the amount of time spent in hospital before and after the CTO between males and females. Data were categorised into five age ranges (Table 1). The groups were compared to see whether one category benefited more or less than the others, using Mann–Whitney *U*-tests as normality of data was not present. Bonferroni correction for multiple testing was applied and *P*<0.05 was considered significant.

**Table 1:** Individuals in each of the five age categories

Age range, years	*n* (%)
0–19	50 (3.2)

20–39	658 (42.2)

40–59	648 (41.6)

60–79	161 (10.3)

80–99	41 (2.6)

## Results

### Cohort and exclusions

A total of 1610 individuals were subject to a CTO in Scotland between 2007 and 2012, and all were available for analysis. Thirty-two were on orders under the Criminal Procedure (Scotland) Act 1995 before CTO application, and it was decided that this legislation added another level of complexity, so these individuals were excluded. Further, 20 individuals had obviously erroneous data relating to their compulsory treatment and length of detention (including either a negative number of days or more than 365 days per year spent in hospital) and were also excluded. Therefore, 1558 individuals subject to a CTO remained for analysis, with approximately 5% of CTOs being new direct from the community.

There were 987 males in the cohort (63%), and the median age was 45 years for men and 39 years for women. Most individuals (*n* = 1358, 87%) had mental illness as a single diagnosis; 11% (*n* = 170) had two or more categories of mental disorder, for instance mental illness and intellectual disability or personality disorder.

**Fig. 1:**
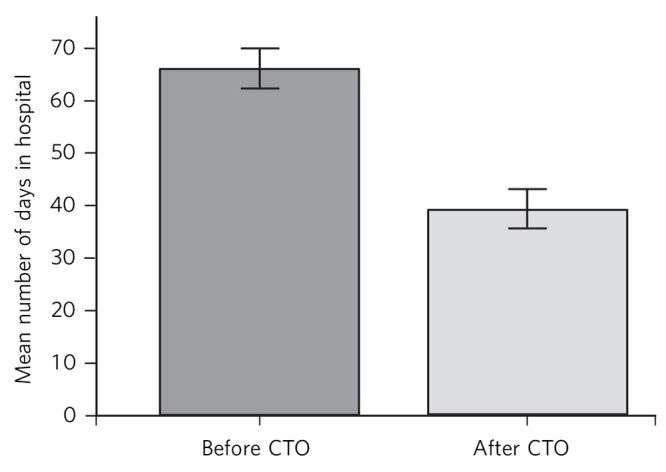
Days in hospital before and after community treatment order (CTO) use. Error bars denote 95% CI.

Comparing mean days in hospital per year before and after a CTO, their number fell from 66.1 (s.e. = 2.0) to 39.3 (s.e. = 1.9) (Fig. 1). A paired-samples *t*-test showed this to be significant (*t*(1558) = 10.5, *P*<0.01).

Further analysis of CTO ‘effect’ on hospital bed days per annum for those on *de novo* CTOs rather than CTOs varied from hospital detention orders revealed that the average number of days in hospital per year after the CTO was in place for *de novo* CTOs was 37, and for those with previous Mental Health (Care and Treatment) (Scotland) Act detention it was 25 (*P* = 0.003 assuming an unpaired *t*-test of samples with equal variance).

In terms of comparing the mean number of days in hospital per year before and after a CTO by gender, for women (*n* = 571) it was 63.6 days before and 38.4 days after the implementation of the CTO, whereas for men it was 67.6 days before and 39.8 days after CTO implementation. A paired-samples *t*-test was done on each sample for both men and women, and indicated a significant reduction in hospital utilisation for patients of both genders (*P*<0.01). An independent-samples *t*-test comparing benefit for males and females gave *P*<0.01, implying men were no more or less likely to benefit from a CTO than women.

**Fig. 2:**
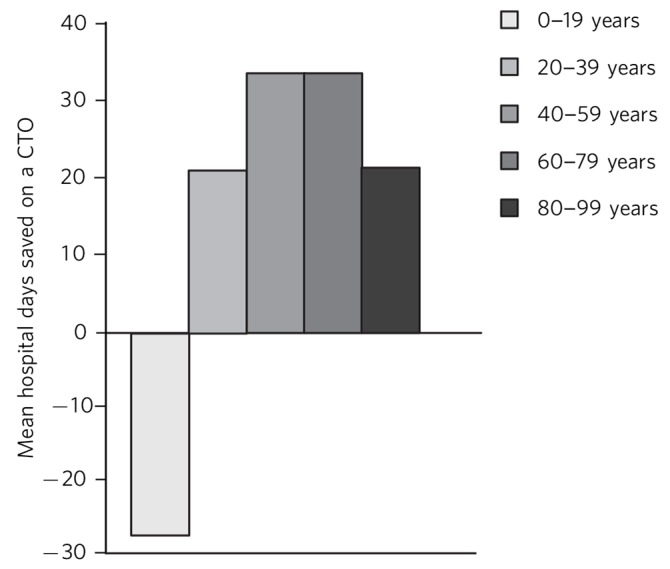
Comparison of mean number of days in hospital per year before and after a community treatment order (CTO) by age.

Figure 2 shows the mean number of days benefit (calculated from mean number of days in hospital per year before a CTO minus mean number of days in hospital per year after a CTO).

Mann–Whitney *U*-tests showed that the age range 0–19 was statistically different from the other groups, except 80–99 (*P*>0.05) (Table 1).

## Discussion

On average, the 1558 individuals subject to a CTO between 2007 and 2012 spent less time in hospital after a CTO than before. Contrary to our hypothesis, mean time in hospital fell from 66 days per year before to 39 days per year after CTO use. This was equally true for men and women. The lower number of hospital bed days for those with previous detentions compared with individuals with new or *de novo* CTOs may suggest that it is individuals with a longer psychiatric history that benefit the most from the imposition of CTOs. Consistent with this, our results also suggest it is middle-aged individuals who have the largest reduction in time spent in hospital after CTO imposition, and that the very young (less than 19 years old) do not appear to be helped by a CTO in terms of reduced hospital stay.

When Light^7^ reviewed CTO usage internationally, she noted the epistemic challenges in assessing their utility and commented that CTOs place responsibilities on both the patient and the healthcare system, binding in service provision. Light also noted that a simple outcome, such as readmission (as measured by the OCTET,^2^ see below) would not necessarily capture important issues such as functionality and quality of life. Kallapiran *et al*^8^ felt that measuring hospital utilisation, as we have here, rather than simply counting the number of admissions might be a better measure of CTO outcome.

### Strengths and limitations

The descriptive results from this study regarding age and diagnosis are remarkably consistent with previous related work by Brown *et al*^9^ using the same database. Strengths of this study include the size of the cohort, being the entire Scottish population subject to a CTO over a 5-year period. Also, hospital admissions were counted over a long time period limiting the bias created by fluctuations in mental illness severity with time (i.e. regression to the mean). Similarly, we corrected for multiple testing and did not go ‘hypothesis fishing’. Although this was not a period of major bed closures and community mental health services were already well developed in Scotland, we cannot exclude the confounding influence of time on our results. Similarly, we cannot simply ascribe the reduction in hospital use to the compulsory treatment mandated by the CTO, as the CTO might bind the individual into a more assertive or effective form of holistic community service. Last, although we examined the effects of age and gender, more detailed analysis (e.g. by specific diagnosis) of who may or may not benefit from CTO use was not possible using the routinely recorded electronic database.

Our findings contradict the previous RCTs of CTO efficacy. The two mature American RCTs^10,11^ are arguably of limited applicability to the UK, given the major differences in healthcare delivery between the UK and the USA. The non-masked OCTET RCT^2^ found no difference in readmissions or overall time spent in hospital when they compared two forms of community compulsion – CTOs and ‘leave of absence’ – even though the length of compulsion between each arm varied (8 *v*. 183 days). OCTET did suffer from a high rate of protocol violations and 20% of those approached to enrol refused, whereas those who were a ‘clear candidate’ for CTO were excluded *a priori*, illustrating the ethical and practical difficulties of conducting randomised studies of potentially uncooperative individuals. The accompanying editorial by Johnson^12^ called for ‘large-scale collation and analysis of routine data already recorded since their (CTO) introduction’, just as we have attempted here.

The paradox between the negative RCT data and the positive case–control studies (as here) and qualitative data deserves discussion. Increased personal autonomy, rates of employment or social stability, and improved quality of life are all potential benefits of CTOs that are difficult to quantify or capture in an RCT design, possibly limiting the generalisability of the RCT findings. Further, despite the inherent bureaucracy and ethical concerns, CTOs remain popular with clinicians and patients' families perhaps in part because they aid in ‘persuading the persuadable’.^13^ It may well be that most individuals are law abiding, and accept the decision of an independent mental health tribunal with regard to the need for compulsory treatment.
